# Dissociation between progression of coronary artery calcification and endothelial function in hemodialysis patients: a prospective pilot study 

**DOI:** 10.5414/CN106830

**Published:** 2011-08-11

**Authors:** Roberto S. Kalil, Michael Flanigan, William Stanford, William G. Haynes

**Affiliations:** Departments of; 1 Internal Medicine and; 2 Radiology, Roy J. and Lucille Carver College of Medicine, University of Iowa, Iowa City, IA, and; 3 Marshfield Clinic, Minocqua, WI, USA

**Keywords:** phosphate binders, atherosclerosis, hemodialysis

## Abstract

Chronic kidney disease profoundly disturbs calcium-phosphate metabolism and predisposes to premature atherosclerosis. Both coronary artery calcification (CAC) and endothelial dysfunction are common in hemodialysis (HD) patients. We hypothesized that a calcium-free phosphate binder would improve endothelial function and delay progression of vascular calcification in HD patients. Methods: This was a randomized parallel-group trial in HD patients comparing lanthanum carbonate (LC) with a non-LC phosphorus binders control group (non-LC) at a 1 : 1 randomization. CAC was obtained at baseline, 6, and 12 months, and endothelial function (brachial artery flow-mediated dilation – FMD) at baseline and 6 months. Results: 13 patients were randomized (LC n = 7 and non-LC n = 6). CAC scores (Log ± SE) at baseline were 7.21 ± 0.62 (LC) and 6.07 ± 0.73 (control). CAC increased in the non-LC group (33 ± 17% and 77 ± 22% at 6 and 12 months), but tended to decrease in the LC group (–10 ± 11% and –2 ± 11% at 6 and 12 months). There was statistically less progression in CAC in the LC group compared to control at 6 (p = 0.002) and 12 months (p = 0.003). There was no difference between groups in FMD (p = 0.7). Markers of inflammation did not change significantly. Conclusion: A slower rate of progression of CAC occurred in the LC group, independent of changes in FMD. This is the first study showing dissociation between progression of CAC and FMD in HD patients. Larger studies are warranted to elucidate the impact of different phosphate sequestration therapies on atherosclerosis in HD patients.

## Introduction

Cardiovascular disease is the main cause of death in dialysis patients [1]. More than 30 years ago, Lindner et al. [2] reported that patients on hemodialysis (HD) suffer from accelerated atherosclerosis. Vascular calcification is highly prevalent in dialysis patients, and may explain their high cardiovascular morbidity and mortality [3]. Vascular calcification has two components, namely arteriosclerosis (thickening and hardening of the arterial walls) and atherosclerosis (the most common type of arteriosclerosis characterized by deposition of fatty acids in the intima leading to plaque formation and obstruction of blood flow); both are present in renal vascular calcifications. Coronary artery calcification (CAC) has been used as a surrogate marker of coronary atherosclerotic disease in the general, predialysis, and dialysis populations [4, 5, 6]. Furthermore, the rate of progression of CAC has been shown to predict coronary events in the general population [7]. Uremia is an inflammatory state, and, as with most of the inflammatory states, it leads to endothelial dysfunction [8, 9, 10]. This may represent another mechanism of accelerated atherosclerosis in dialysis patients [11, 12, 13, 14]. 

Hyperphosphatemia is a well-known predisposing factor for vascular calcification in dialysis patients [15, 16], and has been reported more recently in the general population as well [17]. Poor control of phosphate levels in dialysis patients is associated with higher cardiovascular mortality [18, 19]. Optimal control of hyperphosphatemia may be cardiovascularly protective by attenuating vascular calcification. It is not known whether or not control of hyperphosphatemia with different phosphate binders ameliorates endothelial function or coronary calcification. It is also not known if better endothelial function predicts improved coronary calcification. This prospective, randomized pilot trial examined these mechanisms of atherosclerosis in patients receiving calcium-free, resin-free lanthanum carbonate (LC) compared to non-LC phosphate binders (sevelamer and/or calcium-based phosphate binders). 

## Materials and methods 

### Study subjects

Stage V CKD patients age 18 or older on chronic HD at the University of Iowa Hospitals and Clinics dialysis units requiring a phosphate binder to treat chronic hyperphosphatemia were eligible to participate in the study. Patients being treated with LC were excluded, as were pregnant patients, patients in nursing homes, and patients with poor compliance to dialysis treatments. Eligible patients received the consent form at their dialysis session for their perusal. Two weeks after receiving the consent document, they were asked to consider participating in the study. All patients with history of good adherence to the dialysis treatments were approached. 

### Study design

This was a pilot, prospective, randomized, open-label, parallel-group, single-center study. The study was approved by the University of Iowa Institutional Review Board. 

After signing the consent form, study subjects underwent a phosphate binder wash-out period of 10 days. At the randomization visit to the Clinical Research Unit, patients were randomized to receive LC (Fosrenol^®^, Shire Pharmaceuticals) or to stay under the same binder(s) used prior to the randomization at 1 : 1 ratio. Baseline demographic data including age, gender, race, cause of chronic kidney disease, and dialysis vintage were obtained after consent. A serum pregnancy test was obtained in premenopausal female patients. At randomization (Visit 1), all patients underwent a CAC scan, an endothelial function study by measuring flow-mediated dilatation (FMD) of the brachial artery, and a blood sample draw. At Visit 2, 6 months postrandomization, study subjects returned to the Clinical Research Unit for the same procedures and blood tests performed on Visit 1. At 12 months postrandomization (final study visit), study subjects returned for a coronary calcium scan and blood tests only. 

### Study drug

Dosing of LC was started at 500 mg t.i.d. with meals. Tablets were chewed and taken after food. To achieve target range of phosphorus levels (3.5 – 5.5 mg/dl), dose increments of 250 mg were used as needed. The maximum allowed daily dose was 4,500 mg. Patients were monitored for adverse effects. The LC dose was decreased or temporarily discontinued if suspected adverse effects were present. Patients randomized to stay on the same binders (non-LC group) were started on the same dose after the wash out period. 

### Study procedures

CAC scores: CAC scores were obtained from 16 slice multidetector retrospectively gated noncontrast CT of the coronary arteries. Data containing 3 mm contiguous slices of the entire coronary tree at ± 70% %RR interval were sent for automated scoring. Both Agatston and volumetric scoring were done [20]. The radiologist was blinded to the randomization group of the study. Studies were performed at baseline, 6 months, and 12 months after randomization. Endothelial function: measurements were performed at the Human Cardiovascular Physiology Laboratories of the University of Iowa Institute for Clinical and Translational Science. Endothelial function was assessed by measuring brachial artery diameter during changes in brachial artery flow (FMD). A 13 MHz linear array transducer (AU5, BioSound Esaote) was employed to obtain these measurements. A 5 cm length of the brachial artery was imaged in longitudinal section above the antecubital fossa. Baseline images of brachial artery diameters and Doppler velocities from the center of the vessel were obtained. An occluding forearm cuff was placed 5 cm below the antecubital fossa and inflated to 50 mmHg above systolic pressure for 5 min, and than deflated to induce reactive hyperemia. Brachial artery diameter and velocity were continuously measured during cuff inflation and at 2 min after cuff deflation. Brachial artery dilatation was maximal 1 minute after cuff release and was used as the measure of FMD (endothelium-dependent). The difference in diameter of the brachial artery between baseline and 1 minute after deflation expressed in % was the main parameter for this test. The sonographer performing the studies and analysis was blinded to the randomization of the patient. 

### Laboratory parameters

Laboratory tests were measured at the central laboratories at the University of Iowa Hospitals and Clinics and the analytical laboratory of the Institute for Clinical and Translational Science. Standard biochemical values were obtained at baseline and quarterly after randomization. These included calcium, intact parathyroid hormone (iPTH), alkaline phosphatase, and 25(OH)-D3. Phosphorus levels were obtained at randomization, and then monthly for adjustment of dose of phosphate binder as needed. Biomarkers of inflammation were obtained at baseline, 6 months and 12 months. These markers included high-sensitivity C reactive protein (hs CRP), interleukin-6 (IL-6), asymmetric dimethylarginine (ADMA), and homocysteine. Human IL-6 was assayed by the use of the sandwich enzyme immunoassay technique using a high sensitivity quantikine kit (R&D Systems Inc, Minneapolis, MN, USA). hsCRP concentrations were measured using a sandwich enzyme immunoassay (EIA) from American Laboratory Products (ALPCO Diagnostics, Windham, NH, USA). Plasma ADMA concentrations were measured using a validated HPLC mass spectroscopy method as previously described [21]. Plasma homocysteine concentrations were measured with a validated HPLC method utilizing fluorescence detection as previously described [22]. 

### Statistical analysis

All data are expressed as mean ± SE unless otherwise stated. FMD was compared between LC and non-LC groups using the changes from baseline and 6 months expressed in %. The effect of LC treatment, as compared to non-LC treatment, on the mean change in laboratory measures was tested using linear mixed model analysis for repeated measures. The fixed effects in the mixed model included treatment (LC or non-LC), time, and treatment-time interaction. A significant treatment time indicates that the changes over time differ significantly between the treatment groups. To test specific comparisons of interest (i.e., test for mean change from baseline within each treatment group at 6 months and 12 months; test for between-group difference in mean change from baseline at 6 months and 12 months), a test of mean contrast based on the fitted mixed model was performed. For these tests, a p value < 0.05 was considered statistically significant. For the CAC scores and hs-CRP, log-transformed data were used in the analysis. This was needed to normalize the data distribution of these two measures, which were both left skewed, to satisfy the assumption of normality for the linear mixed model. Mean change at each follow-up time, expressed as mean percent change, was computed by back transformation of the mean change based on the log values. All the statistical analyses were performed using SAS version 9.2 (SAS Institute Inc. Cary, NC, USA). 

## Results 

### Demographics

20 patients signed the consent form. In the LC group, 3 study subjects did not finish the study due to diarrhea, noncompliance with study visits and death due to cardiovascular disease. In the non-LC group, 4 patients did not complete the study due to withdrawing after signing consent or moving to different town or converting to LC by a primary nephrologist due to poor control with other binders. 11 patients completed 3 visits (6 in the LC and 5 in the non-LC group), and 2 patients, 1 in each group, completed 2 visits totaling 13 study participants for data analysis. In an additional 2 patients who developed diarrhea, a temporary discontinuation (1 – 2 weeks) and gradual reintroduction of LC worked well with no relapse of the symptoms. Age ± SD for LC and non-LC groups was 65 ± 9 and 68 ± 9 y, respectively (p = 0.64). Dialysis vintage was 7.5 ± 5 and 3.7 ± 2 y for LC and non-LC, respectively (p = 0.07). Five of the 7 (71%) patients in the LC group were diabetics and 3 of the 6 (50%) control patients were diabetics. 

### Phosphate binders utilized before and after randomization

**Figure 1.**
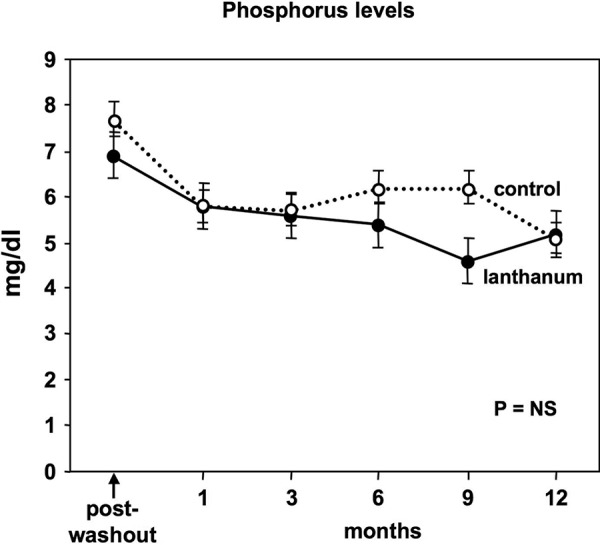
Phosporus levels at randomization day (post-wash out period), at 1,3,6,9 and 12 months.

At the randomization, in the LC group, 4 subjects were taking a combination of calcium-based plus sevelamer, 1 patient was taking calcium only and 1 patient was on sevelamer only. In the non-LC group, 4 patients received a combination of calcium acetate (Phoslo^®^) and sevelamer (Renagel^®^). Two patients were maintained on a calcium-based binder only. After randomization, the daily dose of LC varied from 2,250 to 4,000 mg. In the non-LC group, patients taking calcium-based binders were on a dose range of 2,500 – 4,002 mg daily, and patients on sevelamer were on a dose range of 1,600 – 4,800 mg. Phosphorus levels at baseline (post wash-out period) were 7.0 ± 0.5 mg/dl and 7.7 ± 0.5 mg/dl for LC and non-LC groups, respectively. Phosphorus levels in both groups through the study period were not statistically different (Figure 1). 

### Coronary artery calcification

**Figure 2.**
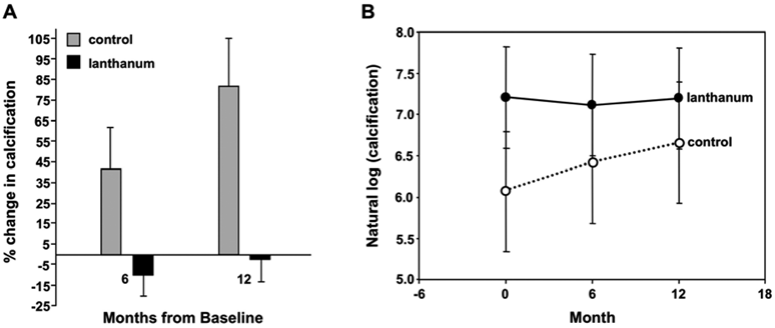
A: Change in CAC LC vs. control: p = 0.02 and 0.003 at 6 and 12 months compared to baseline. B: Coronary artery calcification (log transformed) changes over time, –—— lanthanum; •••••• control.

All participants had detectable CAC at baseline with scores of 2,669 ± 2,723 (182 – 7,083) for the LC group and 1,245 ± 15,70 (48.5 – 1,291) for for the non-LC group. Subjects receiving LC had stabilization of CAC, with a change of –10 ± 11% and –2 ± 11% at 6 and 12 months. In contrast, subjects in the control group exhibited an increase in CAC, with a change of 33 ± 17% and 76 ± 22% at 6 and 12 months (Figure 2A). At baseline CAC scores were not significantly different (p = 0.26). There were significant differences between groups in the change from baseline in CAC at 6 (p = 0.02) and 12 months (p = 0.003). These differences were based on the log-transformed scores. Baseline, 6 and 12 months CAC for LC were 7.2 ± 0.6, 7.1 ± 0.6 and 7.19 ± 0.6, respectively. Baseline, 6 and 12 months CAC for the non-LC group were 6.0 ± 0.7, 6.41 ± 0.7 and 6.66 ± 0.7, respectively (Figure 2B). 

Using the absolute CAC scores to compare the changes in variation over time, the results were as follows: At 6 and 12 months, the LC group had a change (median with 25^th^ – 75^th ^percentile) of –202 (–441 – 38.5), p = 0.56 and 9.2 (–219.7 - 417), p = 0.84. For the non-LC group at 6 months, the change was 229.9 (42 – 859), p = 0.25, and at 12 months 225.8 (68 – 1,017), p = 0.06. Wilcoxon-rank-sum test did not detect significant difference between the two groups in nonlog-transformed scores at 6 months (p = 0.11) or 12 months (p = 0.12). 

### Endothelial function

**Figure 3.**
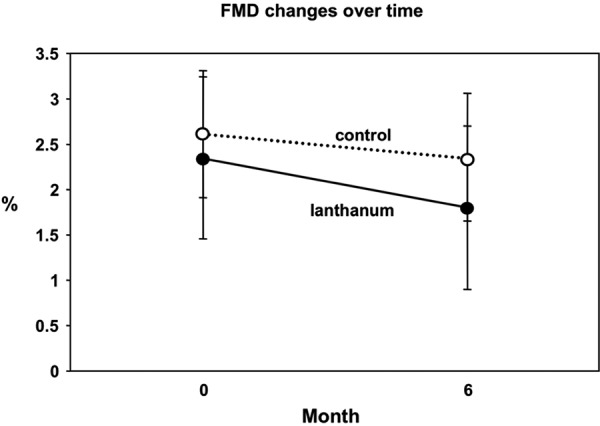
LC vs. control: Flow-mediated dilatation (FMD) at baseline and 6 months. Change in FMD from baseline at 6 months from baseline did not differ significantly between groups (p = 0.77).

FMD was impaired at baseline following phosphate binder wash out. Baseline FMD was 2.3 ± 0.7% and 2.6 ± 0.9% for LC and non-LC, respectively (usually FMD is > 5% in healthy subjects [23]). FMD remained impaired at 6 months despite adequate phosphorus control. At the 6-month visit, no significant changes were observed when compared to baseline, 1.8 ± 0.7% and 2.4 ± 0.9% for LC and non-LC groups, respectively (Figure 3). 

### Laboratory parameters

Phosphorus, calcium, PTH, alkaline phosphatase, homocysteine, and 25(OH)-D3 levels were similar at baseline, 6 and 12 months in both groups (Table 1). Baseline hsCRP levels were higher in the non-LC group, but changes over time were not statistically different between the two groups. Interestingly, there was a reduction in IL-6 of more than 50% from baseline in the LC group, with no change in the non-LC group, but these differences did not reach statistical difference at any timepoint (Table 1). 

## Discussion 

Adequate phosphate control is recommended as a major step towards controlling vascular calcification and improving cardiovascular outcomes in patients receiving chronic HD [24]. The main goal of this study was to examine whether phosphate control with LC compared to calcium-based binders with/without sevelamer would improve endothelial function and/or CAC. Due to the well-established association between inflammation, endothelial dysfunction and atherosclerosis, we hypothesized that an improvement in CAC score would be associated with improvement in endothelial function and markers of inflammation. To our knowledge, this is the first prospective study investigating whether phosphate binders impact vascular calcification, endothelial function, and markers of inflammation concomitantly in dialysis patients. In a diabetic population, Schindler et al. [25] recently reported an improvement in coronary endothelial function, associated with slowed progression of CAC in Type 2 diabetics with glucose-lowering treatment at 1 year. 

We demonstrate, for the first time, that LC decreases the rate of progression of coronary calcification, despite similar control of phosphate levels in both of our groups. All our study patients had detectable CAC at baseline, which was expected considering their dialysis vintage. It is important to note that interscan variability in measuring coronary calcium ranges from 11% to 19% in different series [26, 27]. However, this technical variability would not explain the magnitude of difference observed in our study. In a previous prospective study by Chertow et al. [28], it was demonstrated that dialysis patients receiving a calcium-based phosphate binder exhibited significantly higher CAC scores at 1 year (median increase 25%), compared to patients on sevelamer who had a 5% median increase, under the same phosphate control in both groups. This study did not address endothelial function or markers of inflammation. 

Ours is the first study to compare LC to calcium-based phosphate binders with/without sevelamer. Ideally we would have compared LC with monotherapy, either calcium or sevelamer alone. However, in our patient population, many patients require a combination of binders to achieve adequate phosphate control, and due to cost and adherence reasons, calcium-based binders are the primary choice for most of the patients. Other investigators, such as Hutchison and Laville [29] also demonstrated that a significant fraction of dialysis patients, up to 40%, require more than one binder to achieve adequate control. Non-LC patients received a mix of calcium-based binder with/without sevelamer. It is possible that, in part, the worsening calcification scores observed in the control group were due to the use of calcium in all patients. There are two factors that may have underestimated the possible benefit of LC on CAC. First, there were more patients with diabetes mellitus in the LC group. It is known that diabetics have a higher rate of CAC, even in the predialysis stages [30]. This would have predisposed to worsening CAC in the LC group. Second, the higher baseline CAC in the LC group would predict a higher rate of progression based on data showing that asymptomatic patients with higher CAC at baseline have more marked progression of CAC [31]. 

Interestingly, despite the attenuation in coronary calcification, no parallel improvement in endothelial function was observed. Virtually all known traditional coronary artery disease risk factors lead to endothelial dysfunction. The endothelium is a major target of multiple inflammatory pathways causing atherosclerotic plaque formation [32, 33]. Uremia is known to cause endothelial dysfunction [9]. It was somewhat surprising to find that progression of CAC was slowed independently of endothelial dysfunction or markers of inflammation after achieving a similar phosphate level in both groups. Also, one reason that FMD might not have changed is that structural changes in the arteries may not have reversed by 6 months. This dissociation between changes in CAC and endothelial function has not been demonstrated before in dialysis patients. This observation leads us to speculate that there are nontraditional mechanisms of progression of atherosclerosis in dialysis patients. It is interesting that FMD remained nearly unchanged despite correction of hyperphosphatemia. Our data cannot clearly explain the reasons for this dissociation. It is possible that uremia is the leading cause of endothelial dysfunction in our patients, and that other metabolic factors such as bone mineral disorder will not change vascular function in the presence of advanced uremia, even if adequately controlled. 

ADMA is a potent inhibitor of endogenous nitric oxide synthesis (eNOS) and is found in elevated levels in CKD patients [34, 35]. It is also associated with cardiovascular mortality in HD patients and associated with endothelial dysfunction [36]. ADMA levels did not change over time in both of our groups. Markers of inflammation (such as hsCRP and IL-6) are known to be elevated in HD patients and linked to cardiovascular disease and mortality. We found that hsCRP levels were higher in the non-LC group at baseline. Whether or not this difference had an impact in the progression of the CAC is not known. Another marker of inflammation, IL-6, had a near 50% reduction from baseline for the LC group and no changes in the non-LC group, but these differences did not reach statistical differences. We saw no changes in homocysteine, another marker of cardiovascular mortality in HD patients [37]. 

### This study had several limitations

1. In dialysis patients, CAC is multifactorial, and arterial medial calcifications are highly prevalent, in addition to intimal deposits. Vascular media calcification has a different pathophysiology of the classic intimal plaque formation and possibly a different impact on cardiovascular outcomes in dialysis patients. Although there was a progression of the CAC, one cannot tell if the progression was mainly in the intimal, media or both. However, despite these anatomical differences, London et al. [38] demonstrated an association between arterial media calcification and cardiovascular outcomes.
2. Patients randomized to LC had higher baseline CAC scores. Whether or not the rates of progression of CAC would have differed if both groups had a similar baseline CAC score is not known. In non-HD patients, a study examining progression of CAC revealed that a higher baseline CAC is the most important factor determining higher rates of progression of CAC [31]. There are no published data suggesting that HD patients with higher rates of CAC have different rates of progression.
3. CAC measured with noncontrast CT is an intermediate surrogate marker for atherosclerosis. In the general population, there is a known association between high CAC scores and cardiovascular events [4] because CAC is predominantly found in the intimal layer and correlates better with plaque formation/progression.
4. The sample size was small, although the magnitude of change in FMD (almost none) suggests that even with a larger number of patients, the results would not have been significantly different.
5. We tested FMD as a possible association with changes in CAC along with some markers of inflammation. This assessment is limited to measurement of NO-dependent endothelial function. Additional methods to assess vascular health such as measuring vascular “stiffness” with pulse wave velocity assessment might have shown stronger links to CAC progression.
6. The multifactorial nature of vascular calcification in uremia makes it difficult for a pilot study to draw firm conclusions. The simple avoidance of extra calcium as a binder in the LC group could be a factor in the differences observed here since it is known that high calcium intake leads to higher rates of CAC in the general population.

In large, prospective randomized trials, some atheroprotective interventions such as aggressive lipid-lowering with statin drugs did not translate into protective benefits in dialysis patients [39, 40]. These trials raise the possibility of different mechanisms underlying the progression of vascular disease in HD and atherosclerosis patients and should prompt future mechanistic studies in this high-risk population. 

In summary, we show that chronic dialysis patients have slower progression of CAC under the treatment with LC as a phosphate binder without parallel improvement in endothelial function or markers of inflammation. Future larger studies are needed to continue to explore causes of progression and potential interventions aimed to improve vascular calcification in dialysis patients. Without better understanding of the pathophysiology of progression of coronary artery disease in this patient population, it will be very difficult to explore new therapeutic options to improve cardiovascular outcomes in dialysis patients. 

## Disclosures 

This study was supported through an investigator-initiated study grant (R.K.) from Shire Pharmaceuticals. 

## Acknowledgment 

Dr. Kalil is supported by a grant from the National Heart and Lung and Blood Institute (NHLBI # 5K23 HL08410-02) and by a Clinician Scientist Award from the National Kidney Foundation (NKF). Dr. Haynes is supported by the National Institutes of Health, grant NHLBI P50 HL14388. Portions of this work were presented as a poster at the Renal Week 2009, San Diego, CA, USA. 

**Table 1.** Table 1.

Laboratory test	Baseline		6 months		12 months	
Study Group	LC n = 7	Control n = 6	LC n = 7	Control n = 6	LC n = 6	Control n = 5
Phosphorus^a^	7 ± 0.5	7.7 ± 0.5	5.4 ± 0.5	6.3 ± 0.6	5.2 ± 0.6	5.1 ± 0.6
Calcium^a^	9.8 ± 0.2	9.5 ± 0.3	9.4 ± 0.2	8.9 ± 0.2	8.7 ± 0.3	9.3 ± 0.4
PTH^a^	329 ± 85	321 ± 85	327 ± 85	382 ± 85	420 ± 87	302 ± 120
Alk Phos^a^	97 ± 23	134 ± 25	101 ± 13	100 ± 14	120 ± 21	123 ± 23
25 (OH) D3^a^	26 ± 8	18.3 ± 8	23 ± 8	33 ± 8	29 ± 8	18 ± 8
ADMA^a^	0.53 ± 0.06	0.64 ± 0.07	0.63 ± 0.06	0.61 ± 0.07	0.5 ± 0.07	0.58 ± 0.09
hsCRP (log)^b^	0.9 ± 0.4	2.4 ± 0.4	0.8 ± 0.7	2.4 ± 0.9	0.4 ± 0.6	1.5 ± 0.7
IL6^a^	9.3 ± 1.6	7.3 ± 1.7	7.6 ± 1.6	6.9 ± 1.9	3.9 ± 1.8	7.4 ± 1.9
Homocysteine^a^	28 ± 3.2	22 ± 3.5	28 ± 3.2	21 ± 3.7	21.8 ± 3.9	20 ± 5.2

Baseline, 6 months and 12 months post randomization levels of laboratory parameters. ªp = NS at baseline and 6 and 12 months. p = 0.02 at baseline.
